# Sanitary program to reduce embryonic mortality associated with infectious diseases in cattle

**DOI:** 10.21451/1984-3143-AR2019-0073

**Published:** 2019-10-22

**Authors:** Amauri Alcindo Alfieri, Raquel Arruda Leme, Alais Maria Dall Agnol, Alice Fernandes Alfieri

**Affiliations:** 1 National Institute of Science and Technology of Dairy Production Chain (INCT-Leite), Universidade Estadual de Londrina, Paraná, Brazil.; 2 Laboratory of Animal Virology, Department of Veterinary Preventive Medicine, Universidade Estadual de Londrina, Londrina, Paraná, Brazil.

**Keywords:** bovine, reproduction, IBR, BVD, leptospirosis, vaccination

## Abstract

Among reproductive disorders in dairy and beef cattle worldwide, embryonic mortalities stand out as one of the most frequent. Because of the multifactorial etiology, the clinical and laboratory diagnoses of embryonic mortality causes in cattle are quite complex. Often, infectious causes may account for up to 50% of bovine embryonic mortality rates after 30 days of conception. This review will address the main causes of early and late embryonic mortality, with emphasis on infectious causes and, particularly, those more frequent in the Brazilian cattle herds. In addition, we will discuss ways of controlling and prophylaxis including those related to reproductive and sanitary management, with emphasis on immunoprophylaxis of the three most frequent reproductive infectious diseases in Brazilian dairy and beef cattle herds.

## Introduction

In a recent past of Brazilian cattle breeding, and especially of beef cattle, the embryonic mortality rate was a reproductive parameter that was very little evaluated in the production systems. The lack of information regarding this parameter was general both to part of producers and technicians responsible for the reproduction of the herds. However, with the advent and more recurrent use of reproductive biotechniques, primarily the Fixed-Time Artificial Insemination (FTAI), this important parameter of reproduction in cattle has been evaluated more frequently. The establishment of more standardized FTAI protocols suitable for different geographic regions and different production conditions enabled to compare results that facilitate the identification and quantification of reproductive failures and, in particular, embryonic mortalities. In this review, we approach sanitary programs focused on reducing embryonic mortality associated with infectious diseases. Features of embryonic mortality, related infectious causes, epidemiological profile of infectious reproductive diseases, sanitary programs, vaccination, and biosecurity are addressed.

## Embryonic mortality

In the bovine species, the embryo is defined as the product obtained up to approximately 42-45 days after conception, which refers to the cell differentiation period (Committee on Reproductive Nomenclature, 1972). Consequently, this is the period used to evaluate the embryonic mortality rate in beef and dairy cattle herds. In practice, the return to estrus in an interval longer than 17-25 days reflects the occurrence of embryonic mortality (Ayalon, 1978; Abdalla *et al*., 2017). It should be emphasized that in the analysis of the embryonic mortality rate, one must eliminate fertilization failures.

In cattle, embryonic mortality is a multifactorial event that may involve genetic and environmental factors. Genetic factors are intrinsic to the embryo, and the most frequent are those caused by genetic defects, individual genes and genetic interactions that can lead to chromosomal abnormalities. In cattle, genetic defects may account for up to 20% of embryonic and fetal mortalities (Vanroose *et al*., 2000; Diskin and Morris, 2008).

A range of causes can be included as environmental factors of embryonic mortalities in cattle. Among the several causes, the following stand out: age; climate; hormonal imbalance; uterine environment, among others that can cause physiological and endocrine disorders that can lead to the death of the embryo (Chebel *et al*., 2004; Inskeep and Dailey, 2005; Walsh *et al*., 2011; Abdalla *et al*., 2017; Sánchez *et al*., 2018). In addition, the nutrition factor is also important, especially considering the possibility of postpartum cows present a negative energy balance that may impact the follicular dynamics due to changes in the gene expression of dairy cow granulosa cells at 60 days post-partum, reducing the reproductive performance of this animal category. Therefore, the effects of the negative energy balance may be felt even after the resolution of the problem (Butler, 2003; Girard *et al*., 2015; Lonergan *et al*., 2016; Rani *et al*., 2018). Also in regards to environmental factors, we will highlight the infectious causes.

Embryonic mortality can also be classified into two types based on the time elapsed after conception. Those mortalities that occur before 15 days post-conception are termed early embryonic mortalities and mortalities of 16 to 42-45 days are termed late embryonic mortalities (Inskeep and Dailey, 2005). Comparatively, early embryonic mortalities are more frequent than the late ones (Dunne *et al*., 2000; Inskeep and Dailey, 2005). In cases of early embryonic mortality there will be no change in the period of the estrous cycle. It means that early embryonic mortalities are accompanied by a return to estrus at regular intervals. Conversely, one of the main clinical features of late embryonic mortalities is the return to estrus at irregular intervals (Silke *et al*., 2002).

## Infectious causes of embryonic mortality in cattle

In this category we can include nonspecific causes, represented by a series of bacteria that can cause ascending infections. That is, these bacteria can be present in the vaginal mucosa itself or else in the penis, in cases of reproduction by natural mating, or in the semen in cases of artificial insemination. Often, these opportunistic bacteria cause inflammation in the uterus, resulting in endometritis, which can render the uterine environment inhospitable to the embryo (Bielanski *et al*., 2000; Vanroose *et al*., 2000; Silva *et al*., 2016; Sheldon and Owens, 2017; Rani *et al*., 2018). The frequency of nonspecific infections in bovine females that result in embryonic mortalities in dairy herds is low (Vanroose *et al*., 2000; Sheldon and Owens, 2017). As cases are sporadic, in general, specific measures for the control and prophylaxis of these infections are rarely adopted.

However, the reproductive tract of the bovine female is susceptible to a series of infectious processes caused by pathogenic organisms specific to the reproductive sphere. Infections can be caused by different classes of microorganisms, such as bacteria, viruses, and protozoa (Tab. 1). Although it is possible, fungal infections are rarely a problem of great magnitude in the reproduction of cattle (Vanroose *et al*., 2000; Givens and Marley, 2008; Walsh *et al*., 2011).

**Table 1:** Frequency of embryonic death associated with different infectious agents in beef and dairy cattle herds.

Microorganism		Disease	Infection		Embryonic death
Class	Species		Transmission	Persistence	
Virus	BoHV-1	IBR	Hematogenous	Viral latency	+++
	BVDV	BVD	Hematogenous	Persistent infection	++
Bacteria	*Leptospira* spp.	Leptospirosis	Hematogenous	Renal carrier	+++
	*Campilobacter* sp.	Campylobacteriosis	Genital	Asymptomatic carrier bull	+
	*M. bovigenitalium*	Mycoplasmosis	Genital	Asymptomatic carrier cow	+
	*U. diversum*	Ureaplasmose	Genital	Asymptomatic carrier cow	+
	*Histophilus somni*	Histophilosis	Hematogenous		+
Protozoa	*Tritrichomonas foetus*	Trichomoniasis	Genital	Asymptomatic carrier bull	+
	*Neospora caninum*	Neosporosis	Vertical	Oocyst	+

BoHV-1: Bovine alfaherpesvirus 1; BVDV: Bovine viral diarrhea virus. +: sporadic; ++: frequent; +++: highly frequent. Source: Vanroose *et al*., 2000; Grooms, 2006; Givens and Marley, 2008; Kumar *et al*., 2011; Almería and López-Gatius, 2013; Gates *et al*., 2013; Sanhueza *et al*., 2013; Michi *et al*., 2016.

Infections caused by specific microorganisms can be venereally transmitted, occurring in cases that the organisms are present in the genital tract of the female or male, and by the hematogenous route, which occurs with those organisms that reach the uterus through the bloodstream (Vanroose *et al*., 2000). The epidemic or endemic presentation of the diseases depends on the geographical region, epidemiological scenario, reproductive management, vaccination and health status of the herds. In epidemiological terms, specific infections of the reproductive tract may present in an epidemic form or, more frequently in Brazil, in an endemic manner. The epidemic form prevails in specific pathogen-free herds, that is, they have never been in contact with the microorganism, either by active infection or vaccination. Since these animals lack the active humoral or cellular response specific to the target microorganism, the tendency is the development of the epidemic form of the infection (Alfieri and Alfieri, 2017). In this form, several animals can simultaneously manifest specific clinical signs of disease. In general, they are symptomatic and therefore easier to identify. However, those animals that had the previous infection or those that were previously vaccinated have specific immunological memory, which means that the infection likely will occur in the endemic form. In the endemic presentation, the clinical problems, primarily the embryonic mortalities, may compromise a smaller number of animals for a longer period of time (Alfieri and Alfieri, 2017).

The most important endemic infectious diseases in Brazil are infectious bovine rhinotracheitis, IBR (caused by *Alphaherpesvirus 1* - BoHV-1), bovine viral diarrhea, BVD (caused by *bovine viral diarrhea virus* – BVDV), leptospirosis (cause by *Leptospira* spp.), vulvovaginitis (caused by *Mycoplasma bovigenitalium* and *Ureaplasma diversum*), campylobacteriosis – caused by *Campylobacter fetus*), trichomoniasis (caused by *Tritrichomonas foetus*), and neosporosis (caused by *Neospora caninum*) (Vanroose *et al*., 2000; Grooms, 2006; Givens and Marley, 2008; Kumar *et al*., 2011; Almería and López-Gatius, 2013; Gates *et al*., 2013; Sanhueza *et al*., 2013; Michi *et al*., 2016; Alfieri and Alfieri, 2017; Fischer *et al*., 2018; Rani *et al*., 2018). However, regardless of the form of presentation, epidemic or endemic, both cause considerable economic losses for both dairy and beef cattle (Alfieri and Alfieri, 2017).

## Animal category in a herd

IBR, BVD, and leptospirosis are the three main and most frequent reproductive infectious diseases in the Brazilian cattle herds (Alfieri and Alfieri, 2017; Fischer *et al*., 2018). Particularly in relation to beef cattle, the serological profile for these three infectious diseases in a Brazilian cattle herd differs considerably due to the animal category. In most herds the percentages of non-vaccinated and seropositive animals for BoHV-1, BVDV, and *Leptospira* spp. increase according to the age of the animals. Therefore, the average percentage of nulliparous (heifers) seropositive for these three microorganisms can be considered smaller than the average percentage of primiparous, which is smaller than the average percentage of multiparous cows (Médici *et al*., 2000; Alfieri and Alfieri, 2017). In other words, the percentage of animals susceptible to field prime-infection in a breeding season is higher in heifers than in primiparous and multiparous cows, meaning that the two categories composed by the youngest animals in the herd are the most vulnerable. Consequently, sanitary management for reproductive infectious problems in beef cattle should focus on both heifers and primiparous, particularly when evaluating a vaccination program.

## Epidemiological profile of infectious reproductive diseases

An action of special importance, particularly directed to the control of reproductive diseases, is to obtain information aiming to define the epidemiological profile of the animals and, mainly, stratifying them according to the animal categories. Serological tests should be performed in a percentage of animals that enable defining the seroepidemiology of IBR, BVD, and leptospirosis, for example. Some tests, including the enzyme-linked immunosorbent assay (ELISA) for viruses are qualitative and enable determining the presence/absence of infection in the herd and/or categories of animals that compose the herd. Other diagnostic tests have additional advantages. This applies to the virus neutralization test, especially because of its simultaneous qualitative and quantitative feature, which means that based on the tritation it is possible to establish the magnitude of antibody titers present in the blood serum. High titers evidence recent infection or viral circulation in the herd (Dubovi, 2013; Lanyon *et al*., 2014; Alfieri and Alfieri, 2017).

Even as distinct epidemiological situations the presence of infectious risk factors can influence the occurrence of reproductive problems (Souza *et al*., 2017). The risk of non-infectious early fetal loss appears to increase under the conditions of intensive management systems (Forar *et al*., 1995; Hanzen *et al*., 1999). The non-infectious risk factors, such as nutritional disorders, management failures, and environmental conditions, isolated or in association with infectious causes, may play important role in changes of the main parameters used to evaluate the reproductive efficiency in cattle herds (García-Ispierto *et al*., 2006, 2007a,b). The technical level used in the reproductive activity can influence the presence, frequency, and intensity of health problems, and can generate negative results in a production unit. Therefore, all possible causes of reproductive failures, including infectious, non-infectious, current and previous herd health status, and local and regional epidemiological features should be carefully considered for the resolution/control of the problems.

Furthermore, although the considerable benefits, the indiscriminate use of the biotechnologies of reproduction can generate undesirable consequences, especially when used without a careful analysis of the risk factors inherent to the management. Some factors may compromise the health of the herd, such as the intensive use of parturition areas, increased animal population density at certain periods, abundance and agglomeration of young animals, which are more susceptible to infections (Pegoraro *et al*., 2018). In these situations, the risks of environmental contamination with the main reproductive pathogens, that can be eliminated at the time of birth, are higher than those in traditional breeding systems and facilitate the spread of infections that compromise the reproductive system of cattle. As well, the purchase and sale of genetic material (semen, oocytes, and embryos) should follow some safety principles (Carvalho *et al*., 2007). When the material is imported/exported, clinical isolation and observation of donors, and serological and microbiological tests should be performed to ensure the absence of relevant diseases. These also include epidemiological surveillance in areas where artificial insemination is practiced with imported semen. With regard to embryos, proper collection, manipulation, and transfer techniques can prevent many pathogens of concern (Rufino *et al*., 2006; Carvalho *et al*., 2007). These are some of the examples of non-infectious risks that might be associated with embryonic mortality and other reproductive failures.

Before decision making regarding the health problems causing embryonic mortalities in bovine females, some issues should be raised, such as i) what is the history of the disease in the region and/or in the herd?; ii) how does the microorganism enter the herd?; iii) how is the infection transmitted?; iv) how does the disease remain or how is it kept in the herd (carriers)?; v) are there vectors?; vi) is there an effective treatment?, vii) is there a vaccine to control and prophylaxis?; viii) if it exists, is the vaccine effective?; ix) when and why to vaccinate?; x) are there other controlling forms? Only with answers to these questions is it possible to design an efficient Sanitary Program to reduce the rate of embryonic mortality in beef or dairy bovine herds.

## Sanitary program

In animal production “Sanitary Program” can be defined as a thematic unit constituted by a set of actions developed aiming to promote and maintain the animal health. Unfortunately, particularly for cattle, it is usual to summarize or confuse Sanitary Program with Vaccination Program. When available, vaccines areundoubtedly one of the main actions to be implemented in a Sanitary Program. However, besides the importance of prophylaxis as good practices in vaccinations, there are other important issues, specially the control of disease risk factors. Vaccination prophylaxis strategies may loss efficiency due to the maintenance of the risk factors within the herds. Therefore, complementary actions that promote or preserve animal health are of fundamental importance to obtain quantitative and, mainly, qualitative increase in the beef and dairy production chains. This is directly associated with the financial efficiency of production chains.

One of the main tools that, when available and depending on the epidemiological profile of infections, should be included in a Sanitary Program is the vaccine. In the context of a Sanitary Program for the control of embryonic mortalities in bovine herds, some vaccines should be used not as an additional and emergency measure, but should form a “Vaccination Program”.

Considering the herd animal density and intensive management of a dairy herd and aiming to reduce the embryonic mortality caused by viruses (IBR and BVD), a “Vaccination Program” should be applied to all the females of the herd, regardless of the animal category they belong. Although most of the vaccine manufacturers indicate annual revaccination, biannual revaccinations can be recommended (Alfieri and Alfieri, 2017). It depends on the monitoring results of some reproductive parameters, as well as the qualitative and quantitative (titration) epidemiological profile of the herd. Similarly, the manufacturers' revaccination recommendation for leptospirosis control is biannual. However, in our personal experience and on the basis of epidemiological profile and, mainly, on the antibody titers of the seropositive animals we recommend quarterly revaccinations. Also in the regards of leptospirosis, in very specific situations such as high-producing beef and dairy herds, the therapeutic use of antibiotics may also be prescribed in addition to vaccine prophylaxis in order to reduce the time to control the infection in the herd (Alfieri and Alfieri, 2017).

Commercial vaccines against most of the infections associated with bovine embryonic mortality are available in Brazil. As the epidemiological profile of these infections varies considerably, “Vaccination Programs” should consider the individual variations of each herd. It is important to consider the nutritional management with body score analysis when defining a Vaccination Program, since optimal nutrition is important to enhance immunity and mount an appropriate response to vaccination. Therefore, sufficient protein, energy, minerals, and vitamins are all required to develop and maintain a strong immune system (Berge and Vertenten, 2017). As well, the type of reproductive management should also be considered in the establishment of a Vaccination Program, since pre-breeding vaccination program may improve the health of cows by preventing BVD, trichomoniasis, campylobacteriosis, and leptospirosis (Daly, 2006; Vasconcelos *et al*., 2017), for example. Vaccinating cows against BoHV-1, BVDV, and *Leptospira* spp., particularly when both doses are administered before AI or FTAI improve cow reproductive performance (Pereira *et al*., 2013; Vasconcelos *et al*., 2017). Therefore, a pre-breeding vaccination program aims to increase the chances the cow will breed and ultimately deliver a calf; help the cow become pregnant early in the breeding season; and protect the calf from becoming persistently infected with BVD (Campbell, 2011).

However, in general, infections with BoHV-1, BVDV and *Leptospira* spp. are widely disseminated in Brazilian beef cattle (Junqueira and Alfieri, 2006). Thus, these reproductive infectious diseases are more frequently manifested as endemic and only sporadically as epidemic. As mentioned earlier, in most of the herds, the infection rates among females of different categories vary considerably. It is observed a decreasing percentage rate of seronegative animals and, consequently, more susceptible animals in the nulliparous, primiparous, and multiparous female categories (Junqueira and Alfieri, 2006).

Another important aspect inherent to the pathogenesis of these three bovine reproductive infectious diseases is the tendency to chronicity. Throughout evolution, the etiological agents responsible for these infections have developed strategies to remain in the herds (Alfieri and Alfieri, 2017). BoHV-1, by means of viral latency mechanism (Nandi *et al*., 2009), is able to maintain the viral genome in the nucleus of infected cells, as provirus. Under this viral condition, the infected animal may remain without clinical signs of infection for a long period (Nandi *et al*., 2009). Eventually, the viral latency is broken and the infected animal may present clinical manifestations accompanied by viral re-excretion, perpetuating the infection in the herds (Jones and Chowdhury, 2007; Biswas *et al*., 2013). Regarding BVDV, this viral agent is able to persistently infect (PI) calves, which eliminate high BVDV titers throughout their productive lives, contributing to perpetuate the infectious process in a herd (Hamers *et al*., 1998; Moennig and Becher, 2018). Finally, bovine leptospirosis is considered a chronic infection since most of the times it evolves to chronic kidney disease. The clinical features of the infection, such as the immunological exclusion and intermittent bacterial shedding, make the infection control an important challenge in the herd, especially the serovar Hardjo, which is the most adapted to the bovine species (Adler, 2015; Balamurugan *et al*., 2018).

Concerning the two viral infections (IBR/BVD) this epidemiological feature has a very important practical implication. Depending on the rate of previously infected and, consequently, seropositive animals, in the category of multiparous females the decision to vaccinate or not will depend on a cost-effective analysis of the vaccination. If the decision is vaccinating, a single vaccination dose in the animals of that category is enough, since it is likely that these animals have active immunological memory to these viral agents (Van Drunen Little-van den Hurk, 2006). Additionally, also considering the immunological aspects related to a previous infection, the period between vaccination and artificial insemination may beshorter, and may even adapt to the management on day zero of the FTAI (Daly, 2006; Vasconcelos *et al*., 2017). However, in the categories of more susceptible females, such as nulliparous and primiparous, which have lower rates of seroconversion, the ideal is that before the mating season the animals receive two doses of vaccine with a minimum interval of 21 days between doses (Aono *et al*., 2013; Pereira *et al*., 2013). Also in this situation the second dose, for management reasons may coincide with the day of initiation of the FTAI protocol.

## Vaccination *vs*. reproductive performance of cattle

Hundreds of scientific articles published in peer-reviewed and indexed journals that are available in relevant scientific databases, including Web of Science, PubMed, CAB Abstracts, and others analyzed different variables regarding the vaccination of beef and dairy cattle to control reproductive infections, such as IBR and BVD. These several articles were found by the authors of two meta-analysis based-studies aimed to evaluate the results of vaccination against BoHV-1 and BVDV on the reproductive performance of cattle herds (Newcomer *et al*., 2015; Newcomer *et al*., 2017). In these studies, variables such as the type of reproductive management, body score, herd size, vaccination and/or revaccination program, infection epidemiology in the herds (rate of seropositive animals and antibody titers), type of vaccine (monovalent, polyvalent, inactivated, attenuated), among other less studied aspects were analized. Although the results considerably vary, most studies conclude that vaccination increases the reproductive performance of herds (Pospísil *et al*., 1996; Grooms, 2004; Ficken *et al*., 2006; Zimmerman *et al*., 2007; Aono *et al*., 2013; Pereira *et al*., 2013; Ridpath, 2013; Newcomer *et al*., 2015; Newcomer *et al*., 2017).

Newcomer *et al*. (2015) evaluated the impact of BVDV vaccination on three outcomes in cows: risk of fetal infection, abortion risk, and pregnancy risk. Forty-six studies in 41 separate papers matched the inclusion criteria. The analysis revealed a decrease in abortions of nearly 45% and a nearly 85% decrease in fetal infection rate in cattle vaccinated for BVDV compared with unvaccinated cohorts. Additionally, pregnancy risk was increased by approximately 5% in field trials of BVDV vaccinations. This meta-analysis provided quantitative support for the benefit of vaccination in the prevention of BVDV-associated reproductive disease.

The meta-analysis study developed by Newcomer *et al*. (2017) comprised the analysis of 15 experiments in 10 manuscripts involving more than 7,500 animals. The aim of this meta-analysis was to determine the cumulative efficacy of BoHV-1 vaccination to prevent abortion in pregnant cattle. Risk ratio effect sizes were used in random effects, weighted meta-analyses to assess the impact of vaccination. A 60% decrease in abortion risk in vaccinated cattle was demonstrated. This meta-analysis provided quantitative support for the benefit of BoHV-1vaccination in the prevention of abortion.

## Brazilian vaccination based-studies

In the central-western region of Brazil, Aono *et al*. (2013) evaluated the reproductive efficiency of 16 herds of beef cattle, of which 13 herds did not vaccinate and three herds were regularly vaccinated for IBR, BVD, and leptospirosis. All animals were submitted to the same FTAI protocol and the pregnancy rate was determined by transrectal ultrasonography at 30 and 120 days post-FTAI. The mean rate of pregnancy loss was significantly lower in the animals of the vaccinated herds when compared with the mean rate of embryonic loss observed in animals from unvaccinated herds. Concerning the category of cows, authors also observed a reduction in the embryonic mortality at 30 and 120 days post-FTAI in vaccinated and non-vaccinated primiparous cows.

Also in the Midwest Brazilian region, the effect of vaccination against IBR, BVD, and leptospirosis was evaluated from six commercial herds of beef cattle. From a total of 1,968 cows, 953 were vaccinated and 1,015 were not-vaccinated. The body score was similar for both groups. The reproductive management and diagnosis of gestation were performed as in the previous experiment. The pregnancy rate was significantly higher in the vaccinated group at both 30 and 120 days of gestation. In the group of primiparous cows there was a significant reduction in embryonic mortality. However, vaccination had no effect in the multiparous cow group (Aono *et al*., 2013).

Alternative vaccination schemes for IBR, BVD, and leptospirosis were also compared. In this experiment, the influence of the day of the first vaccination in relation to the initiation of the FTAI protocol on the pregnancy and pregnancy loss rates in primiparous Nelore cows was analyzed. Two groups of vaccinated animals were constituted. In the first group (pre-vaccinated) the first dose of vaccine was administered 30 days before the initiation of the FTAI protocol and the second dose coincided with the initiation of the protocol. The second group of cows received the first dose on the day of initiation of the FTAI protocol and the second dose 30 days after the end of the protocol. There was an effect of the vaccination scheme used on the pregnancy rate at 30 and at 120 days, being higher in the pre-vaccinated group. However, the vaccination protocol had no effect on the rate of pregnancy loss or embryo mortality (Aono *et al*., 2013).

In a series of four experiments, Pereira *et al*. (2013) also evaluated the effect of different vaccination schemes against IBR, BVD, and leptospirosis in dairy herds of Minas Gerais and Paraná states. The four studies involved the total of 3,640 Holstein or Gir x Holstein lactating cows. All the animals of each study received two doses of vaccines, which were administered in different periods based on the beginning of the FTAI protocol. The results showed that pregnant rates were higher in the groups that received the two doses of vaccine before the time of the FTAI in relation to the control group. As well, pregnancy rates were higher in comparison with the group that received thefirst dose of the vaccine at the moment of FTAI. Therefore, it was concluded that when both doses of vaccines are administered prior to AI there is improve of the reproductive efficiency in dairy production systems.

## Biosecurity

Currently in Brazil, the concept of biosecurity is easily associated with animal health involving the pork and poultry production chains, particularly due to the higher animal density in these both production systems. In Brazilian cattle breeding, the concepts of biosecurity are still very little used. However, we have recently observed in dairy cattle the beginning, albeit timid, of the application of some standards of biosecurity, particularly in herds with high genetics and production. Nevertheless, in the vast majority of Brazilian beef cattle, this important action for the control and prophylaxis of infectious diseases is still neglected.

Important actions must be implemented and regularly monitored in order to increase the biosecurity of the herds. Some are easier to be implemented, while others are more complex. Even at reduced percentages, some of these actions can change production costs, while in others there is practically no additional cost.

Biosecurity can be divided into two types, external and internal. External biosecurity is related to measures aimed at preventing the entry of pathogens into the cattle herd. Measures related to this type of biosecurity include quarantine before the introduction of newly acquired animals, and transit restriction of vehicle, person, and animals. Meanwhile, internal biosecurity is related to the decrease in the chance of transmission of pathogens present within the same cattle herd. Measures should be taken to clean and disinfect the installation, to provide adequate facilities according to the different age groups, to separate diseased animals (isolated facilities), to control rodent and other synanthropic animals, to perform correct disposal of dead animals avoiding the transmission of infectious agents, and to promoted the animal welfare (Sarrazin *et al*., 2014; Sahlström *et al*., 2014; Pegoraro *et al*., 2018).

## Conclusion

Depending on the epidemiology of BoHV-1, BVDV and *Leptospira* spp. infection in a cattle herd, especially in certain categories of females of the herd, as well as the vaccination scheme used for the control and prophylaxis of these reproductive diseases the use of vaccines can contribute considerably to the increase the pregnancy rates and reduce embryonic mortality rates in both the Brazilian beef and dairy cattle herds.
